# Prediction of Exercise Capacity and Training Prescription from the 6-Minute Walk Test and Rating of Perceived Exertion

**DOI:** 10.3390/jfmk6020052

**Published:** 2021-06-14

**Authors:** John P. Porcari, Carl Foster, Maria L. Cress, Rachel Larson, Hannah Lewis, Cristina Cortis, Scott Doberstein, Marc Donahue, Andrea Fusco, Kimberly Radtke

**Affiliations:** 1 Department of Exercise and Sport Science, University of Wisconsin-La Crosse, La Crosse, WI 54601, USA; jporcari007@gmail.com (J.P.P.); mcress3381@gmail.com (M.L.C.); rshaw46@gmail.com (R.L.); brownhann1605@gmail.com (H.L.); sdoberstein@uwlax.edu (S.D.); kradtke@uwlax.edu (K.R.); 2 Department of Human Sciences, Society and Health, University of Cassino and Lazio Meridionale, 03043 Cassino, Italy; c.cortis@unicas.it (C.C.); andrea.fusco@unicas.it (A.F.); 3 Mayo Clinic Health System, La Crosse, WI 54601, USA; donahue.marc@mayo.edu

**Keywords:** physical fitness, internal load, RPE, performance

## Abstract

Walking tests, such as the 6-min walk test (6MWT), are popular methods of estimating peak oxygen uptake (VO_2_peak) in clinical populations. However, the strength of the distance vs. VO_2_peak relationship is not strong, and there are no equations for estimating ventilatory threshold (VT), which is important for training prescription and prognosis. Since the 6MWT is often limited by walking mechanics, prediction equations that include simple additional predictors, such as the terminal rating of perceived exertion (RPE), hold the potential for improving the prediction of VO_2_max and VT. Therefore, this study was designed to develop equations for predicting VO_2_peak and VT from performance during the 6MWT, on the basis of walking performance and terminal RPE. Clinically stable patients in a cardiac rehabilitation program (*N* = 63) performed the 6MWT according to the American Thoracic Society guidelines. At the end of each walk, the subject provided their terminal RPE on a 6–20 Borg scale. Each patient also performed a maximal incremental treadmill test with respiratory gas exchange to measure VO_2_peak and VT. There was a good correlation between VO_2_peak and 6MWT distance (*r* = 0.80) which was improved by adding the terminal RPE in a multiple regression formula (6MWT + RPE, R^2^ = 0.71, standard error of estimate, SEE = 1.3 Metabolic Equivalents (METs). The VT was also well correlated with walking performance, 6MWT distance (*r* = 0.80), and was improved by the addition of terminal RPE (6MWT + RPE, R^2^ = 0.69, SEE = 0.95 METs). The addition of terminal RPE to 6MWT distance improved the prediction of maximal METs and METs at VT, which may have practical applications for exercise prescription.

## 1. Introduction

Exercise capacity is an important quantitative expression of the ability to perform muscular activity. Specifically, the peak oxygen uptake (VO_2_peak), used when there is no confirmatory test to prove that a single-measured VO_2_peak is equivalent to the classical definition of VO_2_max, and the ventilatory threshold (VT) are well correlated with performance in individuals capable of performing prolonged heavy exercise [1,2,3]. As an integrator of the elements of the Fick equation, VO_2_peak is a strong index of overall cardio-pulmonary function [4], and a very strong predictor of survival in clinical populations [5,6,7,8]. Similarly, VT, although a physiologically complex concept [9], has come to be recognized as a better measure of exercise capacity than VO_2_peak relative to the ability to carry out daily activities [2,3]. It has also been shown to be an important prognostic marker [10,11]. Normally, cardiopulmonary exercise testing (which is technically demanding) [12] is required to measure VO_2peak_ and VT. directly However, cardiopulmonary exercise testing is normally only conducted for the purpose of exploring the differential diagnosis of dyspnea or to determine if there is a multi-organ system explanation of exercise intolerance.

Traditionally VO_2_peak and VT are measured in the laboratory, during incremental exercise, on either a treadmill or cycle ergometer, with direct measurement of respiratory gas exchange and/or lactate accumulation [12,13]. However, the technical requirements for performing such evaluations are considerable. A variety of predictive equations for VO_2_peak have been developed [14,15,16]. Although providing reasonable accuracy, they typically require maximal effort exercise testing, which can be challenging and unpleasant for patients and present at least some safety concerns [17]. Accordingly, a number of less technically demanding methods based on real-world ambulatory patterns have arisen [6,7,18,19,20,21,22] which are widely deployed in the fitness and clinical exercise physiology communities. In particular, the 6-min walk test (6MWT) in clinical populations [6,7] and the Rockport 1-mile walking test [21] and the 2-km walking test [22] in fitness populations have shown utility in terms of predicting VO_2_peak. Recent data have also suggested that the VT may be predicted from incremental exercise tests based on simple performance measures [23,24,25,26].

In some of the waking tests used to predict functional capacity, the analytic strategy is based on starting with a population estimate, with that estimate adjusted for walking performance and with further adjustments made on the basis of terminal heart rate (HR) (e.g., smaller reductions in points on the basis of shorter walking times or lower terminal HR). Some equations [21,22] also adjust on the basis of other variables such as age, sex, height, weight, or body mass index (BMI). This solution is attractive and relatively simple, with independent studies demonstrating good estimates of VO_2_peak compared to walking performance alone [23]. 

However, because of the wide interindividual variability in the HR response and maximal HR, and of the profound effect of many medications on the HR response, it would be desirable to find a method of replacing the HR measure in these prediction equations. Eston et al. [20] have suggested that the progression of the rating of perceived exertion (RPE) during incremental exercise can be used as a tool for predicting VO_2_peak. Following this line of thinking, Alamji et al. [23] have suggested that the RPE could also be used to predict VT.

Given that simple walking tests such as the 6MWT are much more assessable to the exercising community than laboratory-based tests, and that most training takes place at intensities just below the VT [3], this study was designed to determine whether data from the 6MWT, with walking performance and terminal RPE as predictor variables, could be used to develop adequate prediction equations for VO_2_peak and VT.

## 2. Materials and Methods

The subjects for the study were 63 adult volunteers. All were participants in either a Phase II cardiac rehabilitation program or a community-based exercise program, designed for the primary and secondary prevention of cardiovascular disease. Diagnoses for patients were conventional: stable angina pectoris (*n* = 2), post myocardial infarction (*n* = 22), post revascularization surgery (*n* = 18), post percutaneous intervention (*n* = 14), stable heart failure (*n* = 7), and risk factors for cardiovascular disease (*n* = 25). The research protocol was approved by the University of Wisconsin-La Crosse Institutional Review Board for the Protection of Human Subjects (Protocol 13-HB-001, approved 13 December 2013). All subjects provided written informed consent before participation.

Each subject performed an incremental treadmill exercise test to fatigue or to clinical signs and symptoms [13] with continuous electrocardiographic (ECG) and hemodynamic monitoring. A modified Balke type protocol was used, with the treadmill speed individually selected to represent comfortable walking during the first 2-min stage, and with subsequent increments in workload provided by 2% increments in treadmill grade every stage. Respiratory metabolism was measured with open-circuit spirometry using a mixing chamber based metabolic system (AEI Industries, Pittsburgh, PA, USA). The pneumotach was calibrated with a 3-L syringe, and the gas analyzers were calibrated using a reference gas (4% CO_2_, 16% O_2_) and room air. Gas exchange was integrated every 30 s, and the highest 30 s VO_2_ was accepted as VO_2_peak. The VT was identified using both the v-slope and ventilatory equivalent methods [2]. Because the intent of the study was to predict exercise capacity from 6MWT performance, the VO_2_prak and the VO_2_ at the VT (VO_2_@VT) was expressed as maximal Metabolic Equivalents (METs) (Max METs), and METs at the VT (METs@VT), as we felt that this was more clinically relevant and comparable to estimates of exercise capacity based on performance in standard exercise protocols [13,14,15,16]. On a separate day, always within 72 h, each subject performed a 6MWT on a 30-m course with standard conditions and prompting, according to the American Thoracic Society [27]. The distance completed was measured to the nearest meter using laps completed and interpolation between cones placed at 5-m intervals on the walking course. Within 30 s of concluding the 6MWT, the terminal RPE was measured using the classical (6–20) Borg scale [28]. Instructions for using the RPE scale had been discussed with the subject before the start of the 6MWT.

The relationships of 6MWT with Max METs and METs@VT were made using linear regression. Similarly, the relationships of the terminal RPE with Max METs and METs@VT were made using linear regression. Regression equations for predicting Max METs and METs@VT were constructed using multiple linear regression with a stepwise approach, with 6MWT distance entered first and terminal RPE entered second. 

## 3. Results

The characteristics of the subjects are presented in Table 1, with the subjects presented both by sex and as a total group. They were broadly representative of patients in contemporary preventive/rehabilitation programs in terms of age, diagnoses, and medications.

**Figure 1:**
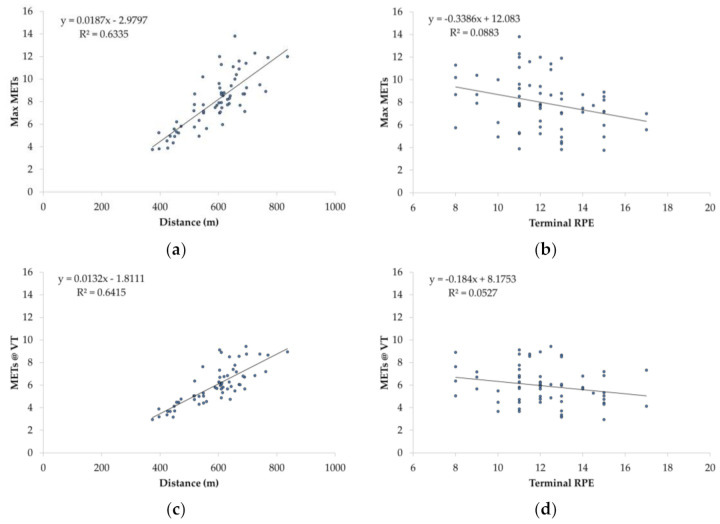
Bivariate relationship between the 6-min walk test (6MWT) distance and the maximal Metabolic Equivalents (Max METs, (**a**)), the terminal rating of perceived exertion (RPE) and Max METs (**b**), the 6MWT distance and Metabolic Equivalents at the ventilatory threshold (METs@VT, (**c**)) and the terminal RPE and METs@VT (**d**).

The bivariate relationships between 6MWT distance versus Max METs and METs@VT, and between terminal RPE vs. Max METs and METs@VT, are presented in Figure 1. There was a strong and significant simple correlation between 6MWT distance versus Max METs (*r* = 0.80) and METs@VT (*r* = 0.80), and a weak but statistically significant correlation between terminal RPE versus Max METs (*r* = 0.30) and METs@VT (*r* = 0.23).

When the 6MWT distance and terminal RPE were combined in a multiple regression equation to predict Max METs, the R^2^ increased from 0.63 to 0.71, with a SEE of 1.3 METs and a standardized residual of 1.0 METs. The prediction equation was:

Max METs = 0.882 + (0.018 * 6MWT m) − (0.308 * RPE)(1)

When the 6MWT distance and terminal RPE were combined in a multiple regression equation to predict METs@VT, the R^2^ increased from 0.64 to 0.69, with a SEE of 0.95 METs and a standardized residual of 0.7 METs. The prediction equation was:

METs@VT = 0.140 + (0.013 * 6MWT m) − (0.161 * RPE)(2)

**Figure 2:**
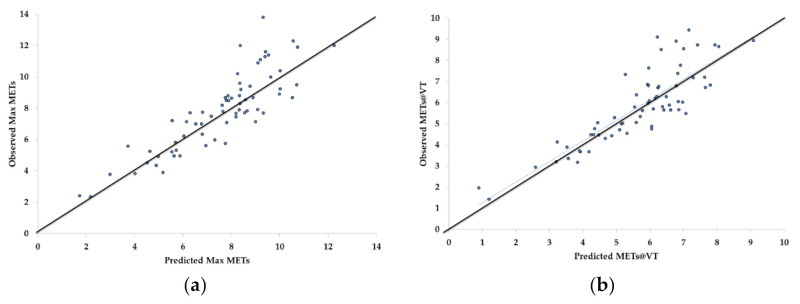

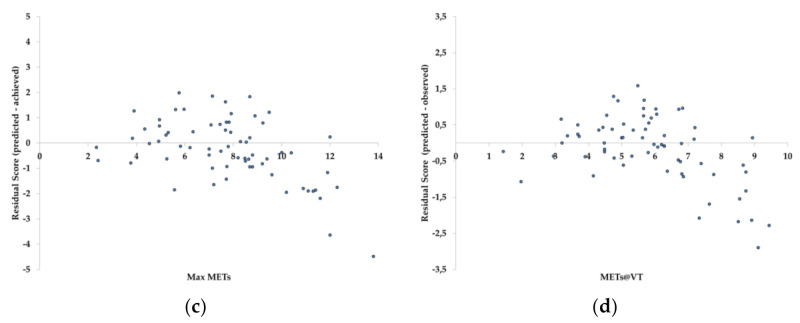
Bivariate relationship between the 6-min walk test (6MWT) + terminal rating of perceived exertion (RPE) prediction equations and observed values for maximal Metabolic Equivalents (Max METs, **a**), Metabolic Equivalents at the ventilatory threshold (METs@VT, **b**) and the standardized residuals for Max METs (**c**) and METs@VT (**d**).

When the derived prediction formulas for Max METs and METs@VT were plotted against the measured Max METs (*r* = 0.87) and METs@VT (*r* = 0.85), there was a strong bivariate relationship for both. The residual scores (predicted-observed) revealed a small value for both Max METS (−0.27 ± 1.24 METs) and METs@VT (−0.14 ± 0.92 METs), with most of the outliers at a relatively higher exercise capacity (Figure 2).

Tabular presentations of Max METs and METs@VT in relation to 6MWT distance and terminal RPE are presented in Table 2 and Table 3. In terms of convenience of use, it is simple to compare 6MWT distance and terminal RPE to derive Max METs and METs@VT.

## 4. Discussion

The main finding of this study was that adding the terminal RPE to 6MWT distance significantly improved the predictability of Max METs. Unique to this study was the ability of 6MWT distance + terminal RPE also to predict the METs@VT, which is a better measure of sustainable exercise capacity [2,9], a powerful emerging measure of prognosis [10,11] and a very useful measure for prescribing exercise [3]. It may also be of particular value in patients with cardiovascular disease who are often on medications that alter the HR response during both exercise testing and training.

The goodness of fit of the equations for predicting Max METs from the 6MWT distance + terminal RPE (R^2^ = 0.71) compares favorably with field tests using the 6MWT distance (R^2^ = 0.42) [6], the Rockport 1-mile Walking Test (R^2^ = 0.78) [21] or R^2^ = 0.71) [23], the Cooper 12-min run-walk Test (R^2^ = 0.80) [19], or with extrapolation of submaximal RPE (R^2^ = 0.85) [20] or (R^2^ = 0.71) [23]. The SEE for all of these predictions equations is on the order of 1.0–1.5 METs. Although the correlation of treadmill protocol time vs. Max METs (e.g., VO_2_max) is typically higher (R^2^ = 0.83–0.94), the SEE is also typically in the range of 1 MET [15,16,29,30]; thus, the simplicity of the 6MWT + terminal RPE is very attractive.

The prediction of METs@VT has more typically been performed using percentages of the maximal power output [25], running speed [23], RPE [23], or as the equivocal stage of the Talk Test [26]. To our knowledge, an approach to estimating the METs@VT in a clinical population with an approach as simple as that used in this study has not previously been reported. Given the importance of the VT in terms of the evaluation of prognosis [10,11] and exercise prescription [3], and the ability of equation-based methods to account for speed and grade equivalents of the METs@VT [31], the ability to predict METs@VT from 6MWT distance + terminal RPE may be of considerable utility.

The workload required to achieve a particular value for the percentage of Max METS (%Max METS), percentage of HR (%HR) reserve, or METs@VT is probably lower than the workload eliciting these markers during exercise testing. Recent research from our laboratory [30] has suggested that “translating” exercise test results to exercise training requires a downregulation of the MET requirement of the workload to ~70–75% of that where a given physiologic response is observed during exercise testing. From that perspective, consider a patient who completes 475 m during the 6MWT, with a terminal RPE of 14. The predicted Max METs would be 5.12 (Table 2), and the METs@VT would be 4.07 (Table 3). Translating to 72% of METs@VT would yield a training intensity of 2.93 METs. Based on the American College of Sports Medicine (ACSM) ambulation equations [31], this workload should be achieved during level-ground ambulation at ~1.13 m*s^−1^ (2.5 mph or 4.1 kph). One would anticipate a RPE during training of ~13 (which has been proposed as the “ideal” intensity for many people) [32] and with comfortable speech still possible [26]. The training load can, of course, be adjusted after the first training bout, but this simple modification of the 6MWT and the simple approach to translating from testing to training [30] suggests a way to define easily the exercise component of the individualized treatment plan for patients in rehabilitation programs.

Another advantage of the 6MWT distance + terminal RPE approach is the ability to make outcome assessments in patients who are mechanically limited to a top walking speed. The 6MWT was originally created for patients with pulmonary disease or heart failure [6,7,26], who often have very limited exercise capacity. In the absence of routine graded exercise testing, and in need of methods for outcome assessment, the 6MWT has more recently been widely used with relatively healthier patients in cardiac rehabilitation programs. These patients often have better exercise capacity and are often constrained by the requirement to only walk during the 6MWT, with a top walking speed unlikely to exceed 2.0 m·s^−1^ (4.5 mph or 7.2 kmh) which requires a VO_2_ of ~16 mL·kg^−1^·min^−1^ (~4.4 METs) [31]. Since common clinical experience shows that many patients can achieve this walking speed more or less comfortably, outcome assessment in rehabilitation programs has been constrained by the use of the 6MWT distance alone. However, in a patient who originally walks 450 m with a RPE of 15, who later walks the same 450 m, but with a RPE of 13, the calculated MaxMETs (4.36 to 4.98 METs) and METs@VT (3.58 to 3.90 METs) improve by 14% and 9%, respectively. Additionally, this follow-up testing can be used to update the exercise proscription. 

Although the present study provides meaningful information, some limitations should be acknowledged. First, this study is a small cohort study. Therefore, future researched should consider a larger sample. Second, the present study did not include a large representation of the heart failure population (i.e., potential heart transplant candidates and those with pulmonary hypertension) in which VO_2_max might have huge significance, in particular, as most of the times it is hard to obtain cardiopulmonary exercise testing in a heart-failure population, and rough estimates based on 6MWT will be of paramount importance.

## 5. Conclusions

The results of this study demonstrate that the simple approach of adding the terminal RPE to the 6MWT distance can improve the estimate of Max METs in patients in rehabilitation programs. Additionally, it can be used to make an estimate of the METs@VT, which is important prognostically [10,11] as a better estimate of sustainable exercise capacity [2] and is highly important prescriptively [3]. Lastly, this approach to estimating Max METs and METs@VT may provide a solution to estimating changes in exercise capacity in patients who are mechanically at the limit of their walking speed capacity. 

## Figures and Tables

**Table 1:** Mean and standard deviation of the characteristics of the subjects.

Characteristics	Men (*n* = 46)	Women (*n* = 17)	Total (*n* = 63)
Age (years)	60.2 ± 9.7	55.4 ± 10.1	59.6 ± 10.0
Height (m)	1.78 ± 0.06	1.62 ± 0.06	1.74 ± 0.09
Weight (kg)	94.4 ± 19.1	65.4 ± 12.7	86.8 ± 21.8
6MWT Distance (m)	583 ± 106	600 ± 87	587 ± 101
6MWT RPE	12.1 ± 2.0	12..3 ± 2.3	12.1 ± 2.1
Max METS	7.8 ± 2.3	8.4 ± 2.4	8.1 ± 2.4
METs@VT	5.8 ± 1.4	6.8 ± 2.0	5.9 ± 1.7
Maximal HR	144 ± 24	161 ± 21	149 ± 24

6MWT: 6-min walk test; RPE: rating of perceived exertion; Max METS: maximal Metabolic Equivalents; METs@VT: Metabolic Equivalents at ventilatory threshold; HR: heart rate.

**Table 2:** Estimate of maximal Metabolic Equivalents in relation to 6-min walk test (6MWT) distance (m) and terminal rating of perceived exertion (RPE). For convenience of presentation, 6MWT distance is incremented by 50 m. However, as the equation is linear, interpolation of intermediate values is appropriate.

								RPE							
Distance (m)	6	7	8	9	10	11	12	13	14	15	16	17	18	19	20
200	2.63	2.33	2.02	1.71	1.40										
225	3.08	2.78	2.47	2.16	1.85	1.54									
250	3.53	3.23	2.92	2.61	2.30	1.99	1.69								
275	3.98	3.68	3.37	3.06	2.75	2.44	2.14	1.83	1.52						
300	4.43	4.13	3.82	3.51	3.20	2.89	2.59	2.28	1.97	1.66					
325	4.88	4.58	4.27	3.96	3.65	3.34	3.04	2.73	2.42	2.11	1.80				
350	5.33	5.03	4.72	4.41	4.10	3.79	3.49	3.18	2.87	2.56	2.25	1.95	1.64		
375	5.78	5.48	5.17	4.86	4.55	4.24	3.94	3.63	3.32	3.01	2.70	2.40	2.09	1.78	
400	6.23	5.93	5.62	5.31	5.00	4.69	4.39	4.08	3.77	3.46	3.15	2.85	2.54	2.23	1.92
425	6.68	6.38	6.07	5.76	5.45	5.14	4.84	4.53	4.22	3.91	3.60	3.30	2.99	2.68	2.37
450	7.13	6.83	6.52	6.21	5.90	5.59	5.29	4.98	4.67	4.36	4.05	3.75	3.44	3.13	2.82
475	7.58	7.28	6.97	6.66	6.35	6.04	5.74	5.43	5.12	4.81	4.50	4.20	3.89	3.58	3.27
500	8.03	7.73	7.42	7.11	6.80	6.49	6.19	5.88	5.57	5.26	4.95	4.65	4.34	4.03	3.72
525	8.48	8.18	7.87	7.56	7.25	6.94	6.64	6.33	6.02	5.71	5.40	5.10	4.79	4.48	4.17
550	8.93	8.63	8.32	8.01	7.70	7.39	7.09	6.78	6.47	6.16	5.85	5.55	5.24	4.93	4.62
575	9.38	9.08	8.77	8.46	8.15	7.84	7.54	7.23	6.92	6.61	6.30	6.00	5.69	5.38	5.07
600	9.83	9.53	9.22	8.91	8.60	8.29	7.99	7.68	7.37	7.06	6.75	6.45	6.14	5.83	5.52
625	10.28	9.98	9.67	9.36	9.05	8.74	8.44	8.13	7.82	7.51	7.20	6.90	6.59	6.28	5.97
650	10.73	10.43	10.12	9.81	9.50	9.19	8.89	8.58	8.27	7.96	7.65	7.35	7.04	6.73	6.42
675	11.18	10.88	10.57	10.26	9.95	9.64	9.34	9.03	8.72	8.41	8.10	7.80	7.49	7.18	6.87
700	11.63	11.33	11.02	10.71	10.40	10.09	9.79	9.48	9.17	8.86	8.55	8.25	7.94	7.63	7.32

**Table 3:** Estimate of Metabolic Equivalents at the ventilatory threshold in relation to 6-min walk test (6MWT) distance (m) and terminal rating of perceived exertion (RPE). For convenience of presentation, 6MWT distance is incremented by 50 m. However, as the equation is linear, interpolation of intermediate values is appropriate.

								RPE							
Distance (m)	6	7	8	9	10	11	12	13	14	15	16	17	18	19	20
200	1.83	1.68	1.53	1.38	1.23										
225	2.10	1.94	1.78	1.62	1.46										
250	2.42	2.26	2.10	1.94	1.78	1.62	1.46								
275	2.75	2.59	2.43	2.27	2.11	1.94	1.78	1.62	1.46						
300	3.07	2.91	2.75	2.59	2.43	2.27	2.11	1.95	1.79	1.63	1.46				
325	3.40	3.24	3.08	2.92	2.76	2.59	2.43	2.27	2.11	1.95	1.79	1.63	1.47		
350	3.72	3.56	3.40	3.24	3.08	2.92	2.76	2.60	2.44	2.28	2.11	1.95	1.79	1.63	
375	4.05	3.89	3.73	3.57	3.41	3.24	3.08	2.92	2.76	2.60	2.44	2.28	2.12	1.96	1.80
400	4.37	4.21	4.05	3.89	3.73	3.57	3.41	3.25	3.09	2.93	2.76	2.60	2.44	2.28	2.12
425	4.70	4.54	4.38	4.22	4.06	3.89	3.73	3.57	3.41	3.25	3.09	2.93	2.77	2.61	2.45
450	5.02	4.86	4.70	4.54	4.38	4.22	4.06	3.90	3.74	3.58	3.41	3.25	3.09	2.93	2.77
475	5.35	5.19	5.03	4.87	4.71	4.54	4.38	4.22	4.06	3.90	3.74	3.58	3.42	3.26	3.10
500	5.67	5.51	5.35	5.19	5.03	4.87	4.71	4.55	4.39	4.23	4.06	3.90	3.74	3.58	3.42
525	6.00	5.84	5.68	5.52	5.36	5.19	5.03	4.87	4.71	4.55	4.39	4.23	4.07	3.91	3.75
550	6.32	6.16	6.00	5.84	5.68	5.52	5.36	5.20	5.04	4.88	4.71	4.55	4.39	4.23	4.07
575	6.65	6.49	6.33	6.17	6.01	5.84	5.68	5.52	5.36	5.20	5.04	4.88	4.72	4.56	4.40
600	6.97	6.81	6.65	6.49	6.33	6.17	6.01	5.85	5.69	5.53	5.36	5.20	5.04	4.88	4.72
625	7.30	7.14	6.98	6.82	6.66	6.49	6.33	6.17	6.01	5.85	5.69	5.53	5.37	5.21	5.05
650	7.62	7.46	7.30	7.14	6.98	6.82	6.66	6.50	6.34	6.18	6.01	5.85	5.69	5.53	5.37
675	7.95	7.79	7.63	7.47	7.31	7.14	6.98	6.82	6.66	6.50	6.34	6.18	6.02	5.86	5.70
700	8.27	8.11	7.95	7.79	7.63	7.47	7.31	7.15	6.99	6.83	6.66	6.50	6.34	6.18	6.02

## Data Availability

The data presented in this study are available on request from the corresponding author.
